# The Importance of REST for Development and Function of Beta Cells

**DOI:** 10.3389/fcell.2017.00012

**Published:** 2017-02-24

**Authors:** David Martin, Anne Grapin-Botton

**Affiliations:** ^1^Service of Cardiology, Centre Hospitalier Universitaire Vaudois (CHUV)Lausanne, Switzerland; ^2^Danish Stem Cell Center, University of CopenhagenCopenhagen, Denmark

**Keywords:** transcription, diabetes, endocrinology, secretion, repressor, embryo, development, pancreas

## Abstract

Beta cells are defined by the genes they express, many of which are specific to this cell type, and ensure a specific set of functions. Beta cells are also defined by a set of genes they should not express (in order to function properly), and these genes have been called forbidden genes. Among these, the transcriptional repressor RE-1 Silencing Transcription factor (REST) is expressed in most cells of the body, excluding most populations of neurons, as well as pancreatic beta and alpha cells. In the cell types where it is expressed, REST represses the expression of hundreds of genes that are crucial for both neuronal and pancreatic endocrine function, through the recruitment of multiple transcriptional and epigenetic co-regulators. REST targets include genes encoding transcription factors, proteins involved in exocytosis, synaptic transmission or ion channeling, and non-coding RNAs. REST is expressed in the progenitors of both neurons and beta cells during development, but it is down-regulated as the cells differentiate. Although REST mutations and deregulation have yet to be connected to diabetes in humans, REST activation during both development and in adult beta cells leads to diabetes in mice.

## Introduction

REST is a transcription factor that represses numerous genes that are essential to the function of beta cells. It is thus actively excluded from beta cells and maintains beta cell genes repressed in other cell types. Similarly, REST is excluded from the majority of neurons, where it was first discovered, and represses neuronal genes in other cell types. We review the discovery of REST as well as its mechanisms of action and targets, mostly based on work with a primary focus on neurons. We also discuss more specific work uncovering how REST repression contributes to triggering beta cell differentiation and function.

## REST, discovery as a repressor of neuronal traits

**Figure 1:**
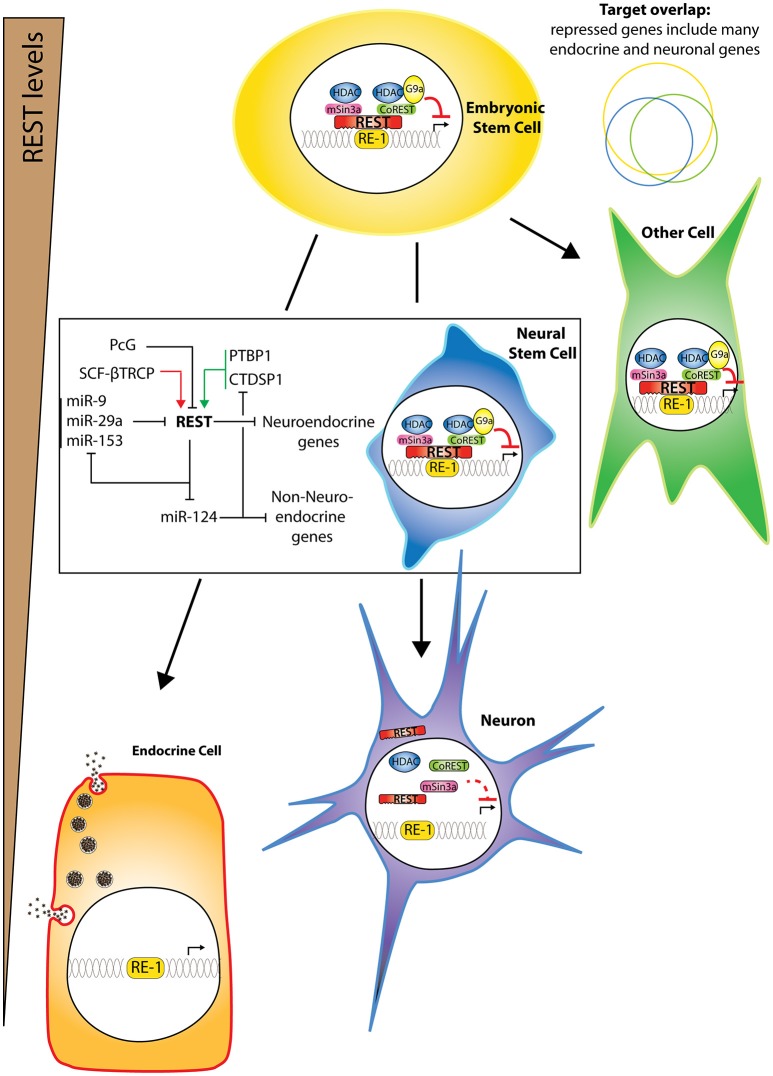
**REST repressor complex and activity in different cell types**. Different cell types express different REST levels. Endocrine cells and neurons express the lowest levels though some neurons can reactivate some REST transcripts at least in pathological scenarios. In all other cell types, there is some binding of REST to its RE-1 target sequence and repressive activity, in conjunction with co-factors. The repressed targets exhibit large but not total overlap between different cell types (with same color code as represented cells). During neuronal differentiation, REST represses neuroendocrine genes, as well as specific miRNAs that are both targets and regulators of REST, thereby forming double negative feedback loops. Imbalanced action of positive (green arrows) and negative (red arrows) regulators of REST stability might trigger a switch toward differentiation.

In 1995, Gail Mandel and David Anderson's groups discovered that the expression of a small battery of genes was restricted to neurons by the recruitment in other cell types of a zinc finger transcriptional repressor to a 21 bp DNA element (Kraner et al., 1992; Mori et al., 1992). This repressor was called RE-1 Silencing Transcription factor (REST) because it binds to a DNA element called repressor element-1 (RE-1) located in the rat *Scn2a2* gene (Chong et al., 1995; Figure 1). In the context of the rat *Stmn2* gene, it was called Neuron-Restrictive Silencer Factor (NRSF) and its target site was named neuron restrictive silencer element (NRSE; Schoenherr and Anderson, 1995). These breakthrough papers, followed by many others reporting the identification of novel REST target genes, described a default pathway whereby the absence of a unique factor (REST) determines part of the gene activity encoding fundamental traits of terminally differentiated neurons (RE-1-containing genes). Target genes of REST were found to be enriched in functions linked to synaptic transmission (Schoch et al., 1996; Bessis et al., 1997; Myers et al., 1998; Lietz et al., 2003; Bruce et al., 2004; Ballas et al., 2005), neurotransmitter signaling (Wood et al., 1996; Bessis et al., 1997; Bai et al., 1998; Myers et al., 1998), ion channeling (Chong et al., 1995; Yeo et al., 2009) and specifically in humans, to learning and memory (Rockowitz and Zheng, 2015), and to neuroprotection and cognitive function (Lu et al., 2014). REST expression was also found in undifferentiated neural progenitors where it prevents precocious expression of the target genes characterizing differentiated neurons (Chong et al., 1995; Schoenherr and Anderson, 1995; Ballas et al., 2005; Figure 1). REST inactivation in mice resulted in embryonic lethality starting after embryonic day (E) 9.5 with ectopic neuronal gene expression in multiple tissues but surprisingly not all (Chen et al., 1998).

Several observations suggest that REST can be present and active in some neurons. Even though it is accepted that REST expression generally decreases during neuronal differentiation (Chong et al., 1995; Schoenherr and Anderson, 1995; Ballas et al., 2005), REST protein (Calderone et al., 2003; Zuccato et al., 2003; Sun et al., 2005; Gao et al., 2011; Lu et al., 2014; Schiffer et al., 2014), and mRNA (Palm et al., 1998; Calderone et al., 2003; Kuwabara et al., 2004; Lu et al., 2014) have been detected in certain mature neurons, especially those of the hippocampus. It is important to note, however, that the genuine detection of REST has been rendered questionable mainly because of a paucity of reliable antibodies or because of a lack of appropriate controls. For example, discrepancies in the molecular weight of immunoreactive bands reported as specific using western blotting may raise several concerns. While the calculated size of REST is of 121 kDa, we, and others, have always observed it around 200 kDa, in experiments including both negative (native beta cell lines) and positive controls (cells overexpressing REST full length). The numerous aspecific bands observed with many commercially available antibodies therefore render questionable observations made using immunostaining without specific controls. In some neurons, alternative splicing of REST mRNA occurs (Palm et al., 1998), giving rise to splice variants of unclear significance such as REST4, which possesses an inserted neuron-specific exon leading to translational frame shift, resulting in a truncated REST protein with four zinc fingers (Palm et al., 1998, 1999). Importantly, disturbance of REST activity in neurons has been linked to several human diseases, as discussed later in this review, suggesting that the control of REST is also important for the maintenance of neuronal function as well as for synaptic plasticity.

In non-neuronal tissues, changes in REST levels induced by pathological conditions may provide a cellular adaptation by affecting the expression of its targets. For example, down-regulated REST expression upon hypertrophic stimulus triggers the cardiac fetal gene reprogramming and correlates with increased smooth muscle cell proliferation in vascular neointimal hyperplasia (Kuwahara et al., 2003; Cheong et al., 2005). In the same spirit, it acts as a tumor suppressor in human epithelial tissues and its de-regulation promotes tumor formation (Westbrook et al., 2005, 2008). It is noteworthy that some of the targets up-regulated in these pathological scenarios are also regulated by REST in the nervous system. However, they are in several instances regulated by REST in a cell type specific manner. For example, NPPA is repressed by REST in cardiac myocytes (Kuwahara et al., 2003) but not in a REST-expressing neuronal cell line (Wood et al., 2003), while CX36 expression can be repressed by ectopic REST expression in pancreatic beta and alpha cell lines but not in a neuronal context (Hohl and Thiel, 2005).

The cell-specificity of REST target occupancy has been resolved at the genome-wide level using ChIP-seq or ChIP-chip experiments, by comparing REST regulatory networks between embryonic stem cells (ESC) and neural stem cells (NSC; Johnson et al., 2008), or between different neuronal and non-neuronal cell lines (Bruce et al., 2009) and is further discussed below.

## Mechanisms of transcriptional repression and epigenetic modifications

REST is a zinc finger protein related to members of the Gli-Kruppel family of transcriptional repressors. The protein contains a cluster of eight zinc fingers, which is required for binding to the RE-1 element, and two repressor domains at the N- and C-terminus (Figure 1). Even though a number of reports showed that the sole ectopic expression of REST is able to mediate target gene repression, REST activity relies on the coordinated recruitment of multiple transcriptional and epigenetic regulators at target loci. This combinatorial recruitment and interplay of co-repressors and chromatin-modifying enzymes defines the complexity and context-dependency of the regulatory landscape controlled by REST. The N-terminus of REST recruits mSin3A (Huang et al., 1999; Grimes et al., 2000), which serves as a scaffold for histone deacetylases (HDACs; Huang et al., 1999; Grimes et al., 2000). The C-terminus interacts with CoREST (Andrés et al., 1999), which also recruits HDACs in addition to the histone H3K4 demethylase, LSD1 (Shi et al., 2004), the H3K9 methyltransferase, G9a (Roopra et al., 2004), the methyl CpG binding protein MeCP2 (Lunyak et al., 2002; Ballas et al., 2005) and BRG-1 and associated factors (Battaglioli et al., 2002; Figure 1). Among others, reviewed in (Ooi and Wood, 2007; Qureshi and Mehler, 2009; Bithell, 2011), REST also interacts with components or effectors of the RNA polymerase II machinery, including the anti-neural small C-terminal domain phosphatase 1 (CTDSP1; Yeo et al., 2005; Visvanathan et al., 2007), Mediator subunits (Ding et al., 2009) and in human cells with the H3K4 demethylase SMCX (Tahiliani et al., 2007) and the corepressor C-terminal binding protein (CtBP; Garriga-Canut et al., 2006; Figure 1). In addition, REST acts in parallel with another key epigenetic system, the polycomb repressive complexes (PRC), which shapes developmental stage-specific regulatory networks by imprinting H3K27 trimethylation at target loci. However, divergent views emerge as to whether the two systems cooperate to drive gene repression at common loci. REST binding motif associates with EZH2-enriched chromatin regions (Ku et al., 2008) and might therefore influence the localization of PRC2-mediated chromatin marks at promoter regions containing RE-1 motifs. However, this crosstalk was not found to translate into transcriptional changes (Arnold et al., 2013). Contrasted observations concluded that REST has context-dependent functions for PRC1- and PRC2-recruitment (Ren and Kerppola, 2011; Dietrich et al., 2012) or that Polycomb and REST complexes direct independent epigenetic modifications controlling early neural fate decisions vs. terminal neural traits acquisition (McGann et al., 2014). Interestingly, the involvement of non-coding RNAs might explain in certain contexts how this cooperative shaping of chromatin states is orchestrated between REST and Polycomb complexes, as it appears that the LncRNA HOTAIR provides a scaffold for collective recruitment of both modifying complexes at the chromatin (Tsai et al., 2010).

## Repertoire of REST-regulated genes

Identifying the repertoire of REST target loci has unraveled an additional level of complexity in the mechanisms dictated by REST to generate cell type-specific and developmental stage-specific gene activity programs (Bithell, 2011). The unusual length of the RE-1 motif has allowed *in silico* identification of putative REST targets across human and murine genomes (Bruce et al., 2004; Mortazavi et al., 2006; Wu and Xie, 2006) and accession to deep-sequencing-associated chromatin immunoprecipitation (ChIP) techniques has increased the number of genes expected to be under REST regulation to the scale of several thousands (Johnson et al., 2007; Otto et al., 2007; Bruce et al., 2009; Rockowitz and Zheng, 2015).

By comparing chromatin occupancy, chromatin modifications, and gene expression in various neuronal and non-neuronal cell types, several studies have provided novel findings. They reveal the existence of different combinations of RE-1 half sites, resulting in a variety of non-canonical RE-1 motifs with varying binding affinities for REST (Otto et al., 2007). Few REST targets were ascribed to non-neuronal functions, such as immune/inflammatory response and cell adhesion, suggesting that REST may not merely be a repressor of neuronal traits (Otto et al., 2007). Another study shows that the number of bound targets decreases as neurons differentiate: among all the targets bound in ESCs and NSCs, 45% are solely bound in ESCs while 50% are shared, and very few are specifically bound in NSC (Figure 1). Many of the ESC-specific REST targets are involved in pluripotency and are commonly targeted by key pluripotency factors OCT4, NANOG, and SOX2, including *Nanog* itself (Johnson et al., 2008). Therefore, REST appears to form an autoregulatory circuit which is connected to the autoregulatory circuit of pluripotency factors. Activating and repressive transcriptional signals thus control ESC pluripotency. When comparing eight neuronal and non-neuronal cell lines, 90% of all REST targets show cell-specificity in REST recruitment. The level of REST protein and the sequence variations in the RE-1 motif govern this modular association of REST to its repertoire of target loci across the different lines (Bruce et al., 2009). REST directly down-regulates a large number of genes at the transcriptional level, but also probably indirectly activates the expression of other genes at the post-transcriptional level via the repression of many noncoding targets (Conaco et al., 2006; Mortazavi et al., 2006; Wu and Xie, 2006; Visvanathan et al., 2007; Singh et al., 2008; Johnson et al., 2009), including several micro RNAs (miRNAs) considered to be brain-specific (such as *miR9, miR124, miR132, miR135, miR139*, and *miR153;* Figure 1). Importantly, REST itself appears to be a predicted target of *miR-153* (Mortazavi et al., 2006; Wu and Xie, 2006), *miR-9* and *miR-29a* (Wu and Xie, 2006). Furthermore, *miR-124* and other neuronal-specific miRNAs target various components of the REST complex, including CTDSP1 and CoREST (Xue et al., 2013). Together, these reports describe a double-negative feedback loop between REST and brain-related miRNAs in controlling neuronal gene expression.

Altogether, these reports integrating transcriptomic and epigenomic data have contributed to a great extent toward the deciphering of the complex mechanisms by which REST shapes regulatory landscapes and controls gene expression in a developmental stage- and cell-specific manner.

## REST is disallowed for normal adult pancreatic beta cell function and maintenance

**Figure 2:**
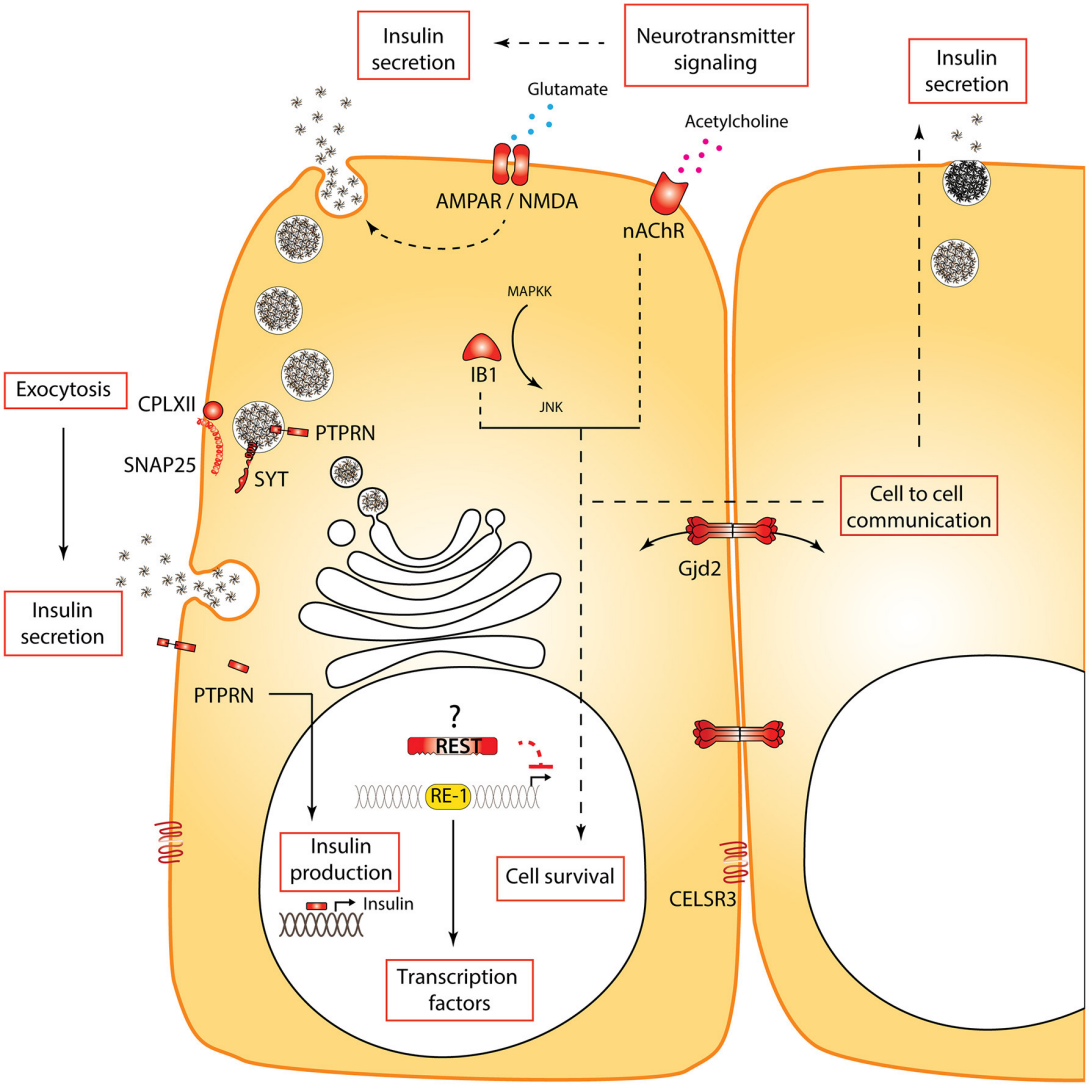
**Processes and specific genes regulated by REST in beta cells**. REST regulates multiple functional processes (boxes) in beta cells. Some non-exhaustive examples of genes and encoded proteins regulated by REST are shown in red. Several of these genes are regulated by REST not only in the functional adult beta cell but also during development. Dotted lines indicate a hypothetical or indirect link. The question mark next to REST indicates that although REST re-expression in beta cells in physiological or pathological conditions was not reported, its re-expression in neurons suggests this may be expected.

Pancreatic beta cells and neurons are derived from different germ layers, the endoderm and ectoderm layers, respectively, yet they share a large number of functional similarities. Beta cells and neurons are both electrically excitable and respond to hormonal stimuli and glucose by depolarization and exocytosis in a process similar to neurotransmitter release. At the molecular level, there are many overlapping patterns of gene expression between the two cell types (van Arensbergen et al., 2010). The shared proteins include enzymes implicated in the synthesis of neurotransmitters, receptors for growth factors and amino acids, neurofilaments, hormones (Atouf et al., 1997), proteins involved in the machinery of exocytosis of synaptic vesicles (Burgoyne and Morgan, 2003), and many transcription factors such as NEUROD/BETA2 (Naya et al., 1995). In 1997, the group of Scharfmann was the first to suggest that, in addition to sharing common transactivators, beta cell lines and neurons are devoid of REST expression. This enables the expression of important functional genes harboring RE-1 motifs including the N-methyl-D-aspartate (NMDA) receptor, the dopamine β-hydroxylase, *Scg10, Synapsin*, and the nicotinic acetylcholine receptor β2 subunit (Atouf et al., 1997; Figure 2). The list of REST-regulated genes expressed in beta cells was further extended with the characterization of RE-1 motifs associated with *Mapk8ip*1/*Ib1* and *Gjd2*/*Cx36* (Abderrahmani et al., 2001, 2004; Martin et al., 2003). MAPK8iP1, identified as a genetic factor associated with type 2 diabetes (Waeber et al., 2000), is a scaffold protein that protects beta cells against apoptosis via the interaction with the c-Jun N-terminal kinase (JNK) signaling pathway (Haefliger et al., 2003) and GJD2, a gap junction-forming protein that controls insulin secretion via cell to cell communication (Haefliger et al., 2003; Head et al., 2012).

Using qPCR and Western blot, we showed that REST was also excluded from adult primary beta cells in mice (Martin et al., 2008, 2015). Importantly, while there are concerns about the specificity of available antibodies (as discussed above in the context of neurons), we provided a western blot analysis including bona fide negative (native beta cell line extracts) and positive controls (beta cells overexpressing murine REST). This allowed to identify the correct immunoreactive band and to unequivocally demonstrate the absence or extremely low levels of production of REST in murine islet cells. Previous transcriptome studies of the pancreas using next generation sequencing on whole islets or purified islet cells showed no or insignificant levels of *Rest* expression in mouse (Ku et al., 2012; Benner et al., 2014) and in human endocrine pancreas (Nica et al., 2013; Blodgett et al., 2015). The advent of single-cell transcriptomic analyses coupled to unbiased *in silico* identification of different cell types recently reaffirmed the concept that Rest is expressed in duct and in acinar cells, while totally absent from human endocrine cell types (Li et al., 2016; Muraro et al., 2016; Segerstolpe et al., 2016). We investigated the functional importance of REST target genes in pancreatic beta cells by ectopic expression of REST in beta cells using RIP-REST transgenic mice. REST mis-expression in beta cells led to impaired glucose homeostasis due to a decrease in both phases of glucose-stimulated insulin secretion. Several defects were noticed that likely accounted for this depressed insulin release: (1) pancreatic insulin content was decreased by half in RIP-REST mice, (2) beta cell counts were 30% lower than in control mice, (3) a selective battery of genes involved in exocytosis of large dense core vesicles, including *Snap25, Syt4, Syt7, Syt9*, and *Cplx2* were under the control of functional RE-1 motif and were down-regulated (Martin et al., 2008; Figure 2). Altogether, our results indicated that besides their implication in insulin secretion, a subset of the RE-1-containing genes may be important for beta-cell turnover. This was confirmed in another line of RIP-REST animals featuring higher levels of REST transgene expression, which developed diabetes due to a dramatic loss in beta-cell mass. Genes relevant to beta cell survival were also found to be under REST control *in vivo*, including *Gjd2, Ib1, Ptprn*, and *Cdk5r2* (Martin et al., 2012; Figure 2). Because of the wide range of naturally occurring combinations of RE-1 half sites in the genome (see above), it is likely that greater expression of REST led to a greater number of bound genes, especially those bearing non-canonical RE-1 motif with suboptimal binding affinity. Mis-expression in adult beta cells using an inducible transgene PDX-tTA; TetO-REST also led to diabetes, as a combined effect of REST mis-expression over both differentiation and maintenance of beta cells (Martin et al., 2015).

Taken together, our work and others' (reviewed in Thiel et al., 2015) show that REST activity needs to be repressed in adult beta cells, qualifying REST as a so-called “disallowed/forbidden” gene (Quintens et al., 2008; Pullen and Rutter, 2013; Lemaire et al., 2016). This classification, however, might be tempered by the fact that the strict definition would imply that *Rest* shows its lowest level of expression in pancreatic beta cells as compared to all other cell types. REST is also absent in other pancreatic endocrine cells as well as most neurons and does thus not strictly qualify the definition. We may need to categorize such genes if they become more numerous. Comprehensive analysis of the chromatin and transcriptional modifications triggered by the mis-expression of REST in adult beta cells would provide valuable insights into the number and role of all REST-controlled genes that constitute this beta-cell-specific sub-signature.

## Role of rest in beta cell development

**Figure 3:**
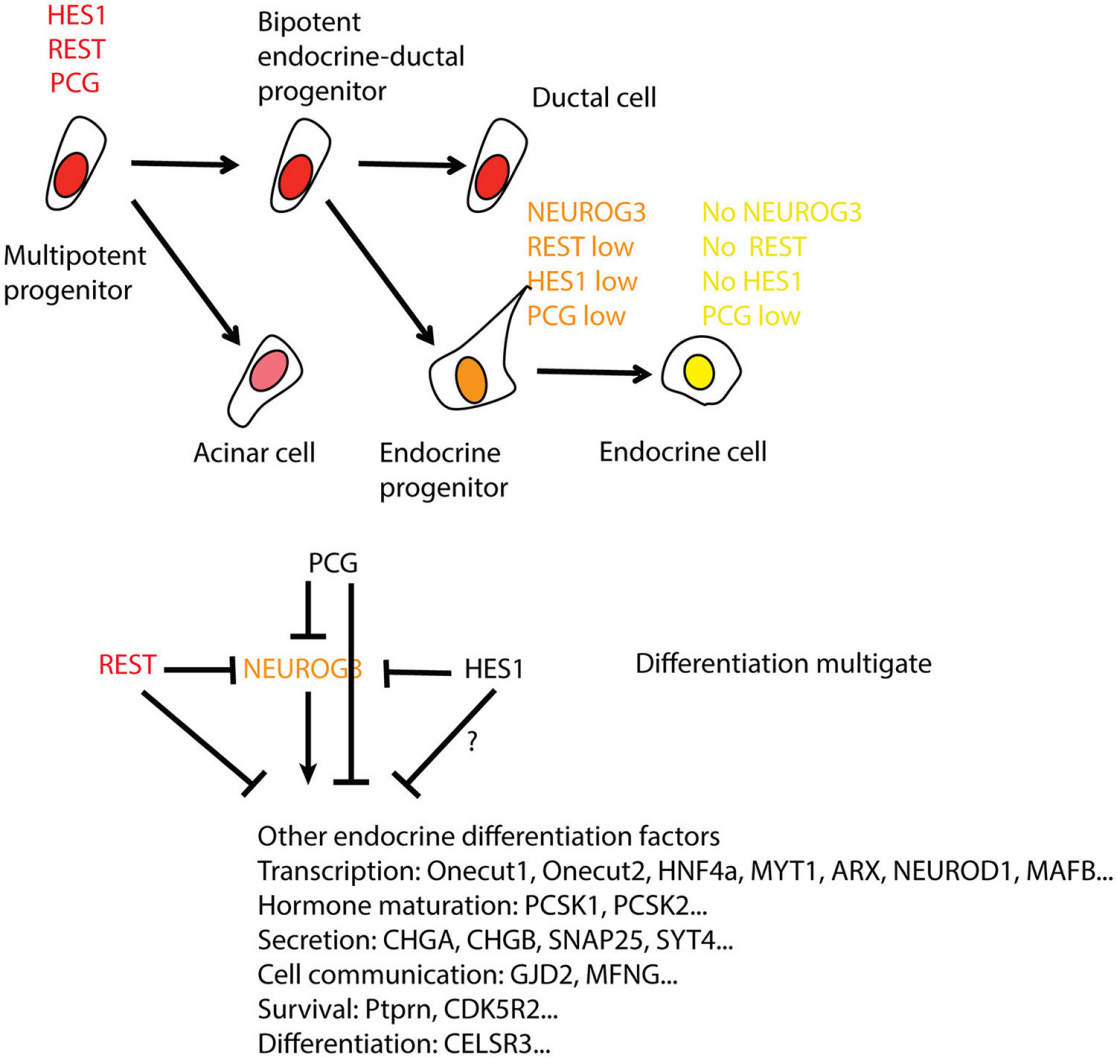
**REST regulation and activity in the differentiation from pancreas progenitors to endocrine cells**. The top panel shows the level of REST/REST activity in the endocrine lineage (red is high, orange medium, and yellow low/absent). REST levels are paralleled by polycomb (PcG) and the Notch target gene HES1. As these transcripts decrease, there is a transient peak of NEUROG3 as progenitors differentiate into endocrine progenitors. The endocrine cells derived from them exhibit low/absent expression of all four. There are multiple parallel blocks on endocrine differentiation by HES1, REST, and PCG at two stages of differentiation, firstly in the progression to endocrine precursors and secondly in their further differentiation into endocrine cells. How endocrine progenitors escape the multiple block to turn on NEUROG3 is unclear. NEUROG3 then promotes the differentiation program. The interactions between REST, HES1, and PCG remain to be deciphered at the level of targets and on their own regulatory sequences.

The exclusion of REST from beta cells (Atouf et al., 1997; Martin et al., 2008), the role of REST as a gatekeeper of neuronal differentiation (Ballas et al., 2005; Gao et al., 2011; Mao et al., 2011; Xue et al., 2013; Love and Prince, 2015) and the similarities between neuron and endocrine cell differentiation suggested that REST may also restrict endocrine cell differentiation during development. Accordingly, we found that *REST* is expressed in progenitors, and is down-regulated in differentiating alpha and beta cells during development (Martin et al., 2015). Several lines of evidence suggest that REST is repressed already in NEUROG3+ endocrine precursors (Figure 3). First, *Rest* transcript is decreased 5.8 times more in beta cells emerging from NEUROG3+ precursors than in young NEUROG3+ cells (http://www.betacell.org/resources/data/studies/view/study_id/3100 and ArrayExpress E-CBIL-48) (Soyer et al., 2010). Second, transcriptome analysis after NEUROG3 *in vivo* gain-of-function at E11.25 (Johansson et al., 2007) shows a 1.9 fold decrease of *Rest* in the pancreatic bud after NEUROG3 over-expression (http://www.betacell.org/resources/data/studies/view/study_id/3733) (Cortijo et al., 2012). The *Rest* gene acquires a Polycomb-mediated H3K27me3 repressive mark after the pancreatic progenitor stage, which can be interpreted as an outcome of the repression of REST expression and/or as a mechanism that ensures its repression (van Arensbergen et al., 2010). ChIP sequencing analysis has detected several REST binding sites in the vicinity of genes that promote endocrine development such as *NeuroD, Neurog3, Onecut1*, or *Hnf4*α (Johnson et al., 2007), which suggests that REST in progenitors represses endocrine differentiation (Figure 3). Hence, gain-of-function in pancreatic progenitors using the inducible transgenic line PDX-tTA; TetO-REST (Martin et al., 2015) severely decreases the formation of NEUROG3^+^ precursors and of differentiated endocrine cells. Moreover, conditional inactivation of REST in pancreas progenitors inhibits the expression of important factors of endocrine differentiation, leading to the formation of endocrine cells in appropriate numbers but in a partially differentiated state. The observation that REST depletion is not sufficient to fully promote endocrine cell formation suggests that there are multiple gates in the differentiation, and that either other repressors or activators are acting parallel to REST. The control over a subprogram is in agreement with what was observed in the context of neuronal differentiation in *Rest*-deficient mice (Aoki et al., 2012) and zebrafish (Kok et al., 2012). In the neural retina, *Rest* deletion in ganglion progenitor cells led to an increased number of retinal ganglion cells, via the up-regulation of crucial regulators of this cell type (Mao et al., 2011). A change in differentiation can be difficult to detect if complex feedback mechanisms are involved. For example, in the adult brain, loss of REST only triggers a transient increase in neurogenesis and depletion of the hippocampal stem cells (Gao et al., 2011).

In the pancreas, NEUROG3, which appears to be a direct target of REST by Chip-Seq is not induced in the knock-out and may be independently repressed by HES1 (Jensen et al., 2000; Figure 3). It is also possible that, in spite of REST binding NEUROG3 regulatory sequences, the chromatin at this locus may not be poised to activation. This scheme is suggested by the observation that REST and Polycomb repressions act in parallel in neuronal differentiation (McGann et al., 2014) as well as in pancreatic beta-cells (van Arensbergen et al., 2010). Indeed, in pancreatic progenitors, PcG marks a wide number of disallowed genes for beta cells, including regulators of alternate developmental fate that will never be activated in the differentiation path, while REST binds in ES cells to genes in which PcG repression disappears in beta cells (Figure 3).

The targets activated upon REST depletion are expected to affect a variety of functions including transcription factors that control differentiation (*Onecut1, Onecut2, Hnf4a, Myt1, Arx, Neurod1, Mafb*), proteins acting in insulin maturation (*Pcsk1, Pcsk2*), insulin secretion (*Chga, Chgb, Syt4*), cell to cell communication (*Gjd2, Mfng*), anti-apoptotic activity (*Ptprn, Cdk5r2*) and differentiation functions (*Celsr3;* Figure 3). Although the subset of REST targets in the pancreas containing *Celsr3* (Jia et al., 2014) and *Syt4* is also under REST repression in the nervous system, many targets are specific to one or the other tissue, suggesting that transcriptional repression by REST functions in a cell type-specific manner, as discussed above. This may be accounted for by the level of REST protein, the sequence variations in the RE-1 motif or the presence of co-factors (Johnson et al., 2008; Bruce et al., 2009).

## REST down-regulation as a switch for neuronal and endocrine differentiation

The examples discussed above illustrate how a tight regulation of REST leads to its expression in most cells and its general exclusion from beta cells and neurons. The mechanisms regulating REST during normal development, adult life and pathological scenarios are still largely enigmatic. Three promoters, six enhancers and two repressor regions with differences of activity in different cell types regulate REST transcription but the transcription factors regulating their activity are unclear (Koenigsberger et al., 2000). The REST gene contains several RE-1 sites, indicating a putative negative autoregulatory feedback on its own expression (Johnson et al., 2007, 2008).

REST is down-regulated as neurons or pancreatic endocrine cells differentiate, but what triggers this is unclear. In the pancreas, it is accompanied by the acquisition of an epigenetic PcG-mediated H3K27me3 repressive mark occurring after the pancreatic progenitor stage, which coincides with the activation of a core beta-cell derepression program (van Arensbergen et al., 2010). During ES cell differentiation into cortical neurons, most of the regulation is initially post-transcriptional while REST transcription is eventually decreased (Ballas et al., 2005). REST abundance and stability are influenced by coordinated kinase, phosphatase, and ubiquitin ligase activities. On the one hand, the CTDSP1 phosphatase activity targets a phosphorylation site in REST and stabilizes it in stem cells. (Yeo et al., 2005; Figure 1). On the other hand, because CTDSP1 expression decreases with differentiation (Visvanathan et al., 2007), the ERK-dependent phosphorylation at the same site, together with the peptidylprolyl isomerase Pin1 activity, promote REST degradation in neural progenitors (Nesti et al., 2014). It appears that phosphorylation of the motif allows recruitment of SCF^βTRCP^, an E3 ubiquitin ligase that is induced during neuronal differentiation and is responsible for REST degradation and subsequent neuronal differentiation (Westbrook et al., 2008). Decreased expression of the deubiquitylase HAUSP during neuronal differentiation further destabilizes REST (Huang et al., 2011). The activity of the REST repressive complex during neuronal differentiation is also tightly controlled at the transcriptional level, and includes a HDAC-dependent regulation triggered by retinoic acid (Ballas et al., 2005). However, the most documented mechanism is post-transcriptional and involves a double negative feedback loop involving miRNAs. REST regulates the expression of miRNAs and is itself regulated by them, including *miR-153* (Mortazavi et al., 2006; Wu and Xie, 2006), *miR-9* and *miR-29a* (Wu and Xie, 2006; Figure 1). *miR-124*, another component of this double feedback loop, has reciprocal activity by inhibiting non neuronal transcripts (Conaco et al., 2006). REST represses *miR-124*, an miRNA that targets various REST components including CTDSP1 and CoREST (Xue et al., 2013). This system of large autoregulatory loops is controlled by another feedforward loop that involves the polypyrimidine-tract-binding (PTBP1) protein, which secures this system by competing at *miR-124* targets (Xue et al., 2013; Figure 1).

It would be of interest to identify which signals, in addition to that directed by PcG complex, dictate REST extinction along the pancreatic developmental pathway.

## Up-regulation of REST activity in neurons promotes neurological diseases. Could pathological REST activation trigger beta cell failure?

Several studies have documented that aberrant induction of REST expression in neurons or progenitors plays a role in neurodegenerative and neurodevelopmental diseases. The example studied in most depth is Rett syndrome, which is a neurodevelopmental disorder caused by mutations in the methyl CpG binding protein 2 gene (MeCP2). This causes an increase in expression of REST and CoREST, and as a consequence the downregulation of their target BDNF (Abuhatzira et al., 2007) and neuron-specific K(+)-Cl(−) cotransporter2 (KCC2; Tang et al., 2016). A few cases of type 1 diabetes have been associated to Rett syndrome, but the number of cases is still too low to determine whether this is mere serendipity or if they were caused by MeCP2 mutations and downstream REST activity (Kurtoglu et al., 2005; Rekik et al., 2010; Akin et al., 2012). De-repression of REST in neurons has also been shown to occur in response to ischemia, leading to down-regulation of REST target genes, notably the GRIA2 subunit of the glutamate receptor regulating Ca^2+^ permeability and miR-29c, that promoted neuronal death (Calderone et al., 2003; Noh et al., 2012; Pandi et al., 2013). Kainic acid, a glutamate analog also promotes REST expression but it is unclear whether this occurs at physiological levels to modulate neuronal function or only upon massive glutamate release (Palm et al., 1998). REST activation has also been reported in the brain of Huntington's disease patients and involves a cytoplasmic sequestering of REST by wild type huntingtin, which is lost with the mutant protein (Zuccato et al., 2003, 2007; Schiffer et al., 2014). Increased REST activity in neurons or progenitor cells is also associated with Down syndrome (Bahn et al., 2002; Lepagnol-Bestel et al., 2009).

Up-regulation of *Rest* mRNA and protein is also a prominent feature of a subset of human glioblastomas and medulloblastomas (Lawinger et al., 2000; Su et al., 2006; Conti et al., 2012; Kamal et al., 2012; Wagoner and Roopra, 2012). These pediatric malignant brain tumors are thought to arise from undifferentiated neural progenitors, and REST may be promoting tumor formation by preventing their differentiation (Su et al., 2006). However, the mechanism underlying REST induction in these tumors remains unknown.

REST induction in the brain may not always be detrimental. REST is promoted by BMPs during astrocyte differentiation from progenitors and is likely to enforce programs divergent from their neuronal sister cells (Kohyama et al., 2010). This appears to be direct and is mediated by a smad-binding site in REST promoter, but whether BMP also promotes REST in other contexts remains to be explored. In addition, it has been proposed that Wnt-driven REST expression increases in aging neurons of the cortex and hippocampus and may play a protective role against Alzheimer's disease and fronto-temporal dementia (Lu et al., 2014).

REST expression is forbidden in pancreatic endocrine cells and violation of this disallowance can provoke diabetes in mice (Martin et al., 2012, 2015). Its up-regulation in humans may also cause or contribute to type 2 diabetes, neonatal diabetes, or maturity onset diabetes of the young. The latter two are caused by mutations in one gene, usually key to pancreatic endocrine development or beta cell biology. Though REST gain-of-function mutations have not yet been reported as causes of monogenic diabetes, the 22 known mutations account for 80% of cases (De Franco et al., 2015). About 20% of the cases therefore remain of unknown genetic etiology.

## REST repression as a tool for reprogramming cells into neurons or endocrine cells

Elucidating the role of REST in development and lineage specification uncovered new roles for this factor in embryonic stem cell (ESC) pluripotency. REST is expressed at high levels in mouse ESC (Ballas et al., 2005) and a series of genome-wide analyses demonstrated that in both mouse and human ESC, REST is a target and partner of multiple factors controlling pluripotency, including NANOG, OCT4, and SOX2 (Boyer et al., 2005; Loh et al., 2006; Wang et al., 2006; Kim et al., 2008). Moreover, a subset of the REST regulatory network is shared with these three factors (Johnson et al., 2008). However, the role of REST as a pluripotency factor remains controversial. Although loss of REST in mouse ESC may decrease the expression of pluripotency factors and their self-renewal capacity via activation of *miR*-21 (Singh et al., 2008, 2012, 2015), other reports showed that REST loss-of-function did not restrict ESC self-renewal or their multiple lineage potential (Buckley et al., 2009; Jørgensen et al., 2009; Yamada et al., 2010). REST may be important, however, for proper timing and acquisition of the primitive endodermal fate by repressing NANOG (Johnson et al., 2008; Yamada et al., 2010).

Recent evidence suggests that depleting non-neuronal cells of REST can promote reprogramming. Fresh mouse cortical astrocytes can be reprogrammed into neurons when transduced with ASCL1 or NEUROG2. However, the conversion of astrocytes cultured for several days is possible only with the inactivation of REST and the activation of either ASCL1 or NEUROG2. Prolonged culture increases the level of H4K20me3 at the *NeuroD4* promoter, a key reprogramming factor, and promotes a local chromatin environment favorable to the repressive complex REST. In these conditions REST outcompetes NEUROG2 in terms of binding to the *NeuroD4* promoter (Masserdotti et al., 2015).

It is not clear whether this change in chromatin at REST loci upon culture is a general principle and whether it can be exploited to reprogram other cell types. Genetic inactivation of REST does not efficiently turn fibroblasts into neurons despite the induction of some neuronal genes, suggesting that additional epigenetic or transcription factors are required (Aoki et al., 2012). A similar strategy applied to generate reprogrammed beta cells had very limited success. Indeed, forced expression of *ShRest/Nrsf, shShh*, and PDX1 in bone marrow-derived mesenchymal stem cells induces several markers of beta cells including insulin, but to <10 fold, which is minimal since these cells express very low levels of beta cell genes (Li et al., 2012). However, shREST was able to trigger neuronal differentiation from MEFs, potentially via a feedforward loop that involves PTBP1 and miR-*124* (Xue et al., 2013). Whether the same paradigm applies to humans is unclear (Xue et al., 2016). In these attempts, one must bear in mind that depleting REST from a cell that does express it may have detrimental effects. A recent model of REST inactivation in neuronal progenitors shows that the absence of REST in proliferative cells that normally express it leads to DNA damage (Nechiporuk et al., 2016). If the affected cells are prevented from dying by p53 inactivation, some develop glioblastoma and primitive neuroectodermal tumor.

Despite the relatively large amount of information regarding the importance of REST in the regulation of the neuronal lineage, the study of REST vis-a-vis endocrine differentiation and beta cell function is in its infancy. Proof of concepts have established that REST must be excluded from beta cells but a global view of its regulation and targets during differentiation, in the embryo, in the early post-natal cells, during aging and in pathological processes is missing. Although it is becoming clear that REST is absent in human pancreatic endocrine cells, a tally of genes regulated by REST would be valuable, as well as information on its expression and role in other endocrine lineages in the intestine. Finally, most research has been restricted to mice but its targets in the human endocrine lineage and its role in diabetes will eventually be of utmost interest.

## Author contributions

DM and AB conceived the review, collected the bibliographic references and wrote the manuscript. Some of the work presented is based on their own experimental investigations.

## Funding

This project was financed by a Novo Nordisk Foundation grant to AB.

### Conflict of interest statement

The authors declare that the research was conducted in the absence of any commercial or financial relationships that could be construed as a potential conflict of interest.
